# Domestic Summary

**Published:** 1839-11-02

**Authors:** 


### DOMESTIC SUMMARY.

*Medical College of Philadelphia.*—The following is the act for incorporating this college, passed at the last session of the Legislature of Pennsylvania, and which has now received the approbation of the executive:

*Sec.* 1. [Contains the act of incorporation, and list of corporators—a number of the most respectable physicians of Philadelphia and its neighbourhood.]

*Sec.* 2. The objects of the corporation hereby created, shall be: to cultivate the science of medicine, and all its collateral branches; to encourage the prolongation of the term of study, and the increase of the extent of preliminary knowledge required of candidates for medical honours; to designate such courses of instruction, as from time to time may be deemed necessary for the advancement of the science, and the elevation of the medical character; and to examine and decide on the qualifications of candidates for medical degrees.

*Sec.* 3. The officers of said college shall be a president, two vice presidents, a corresponding secretary, a treasurer, a recording secretary, and such other officers as shall be provided for by the by-laws; and said officers shall be elected by the members of said corporation, at such times, in such manner, and for such terms as shall be provided for by the by-laws. And said corporation shall have power to enact by-laws for the government, admission, and expulsion of members: *Provided always,* That no organized faculty of professors or teachers shall ever be established by the authority of said college, unless some other collegiate institution or institutions, now or hereafter established within the city of Philadelphia, shall enact laws interfering with the attendance of any medical student upon such course or courses of medical instruction, delivered by authority of such collegiate institution or institutions as said student may prefer or select.

*Sec.* 4. Said college shall have power to grant the degree of bachelor of medicine to any such persons as shall have completed a course of study, similar to that usually required of candidates for the degree of doctor of medicine in other colleges in this state.

*Sec.* 5. Said college shall have power to grant the degree of doctor of medicine to any persons, who shall have fulfilled the requisites hereinafter mentioned, with such others as from time to time may be prescribed by the by-laws.

*Sec.* 6. Each candidate for the degree of doctor of medicine in said college, shall have attained the age of twenty-two years; he shall have pursued the study of medicine for the term of at least three years, under the direction of one or more graduates in medicine; he shall have attended lectures in the city or county of Philadelphia, on each of the following branches, or on such subdivisions thereof as shall be deemed collectively equivalent thereto, delivered by lecturers recognised by said college, and shall have attended the same to the number of courses herein designated, and upon each course for a period of four months: anatomy, general and special, two courses; chemistry, one course; natural philosophy, one course; physiology and pathology, two courses; materia medica and pharmacy, two courses; special therapeutics, two courses; institutes and practice of surgery, two courses; obstetrics, two courses; diseases of women and children, one course; medical jurisprudence, one course. He shall also have pursued at least one course of dissections, under the directions of a teacher recognised by the college; and shall have attended for at least one year the practice of some hospital, containing not less than fifty beds, and in which clinical instruction is given. He shall also produce to the college satisfactory evidence that he possesses a good moral character.

*Sec.* 7. The degrees herein mentioned, shall be granted on such terms and in such manner as shall be prescribed by the by-laws, conformably to the foregoing sections; and all fees, received from persons applying for degrees, shall be distributed and applied in such manner as shall be provided for by the by-laws.

*Sec.* 8. The legislature may, at any time, alter, amend, or repeal the privileges hereby granted.

---

*Means of repressing Hæmorrhage from Arteries.*—Professor Macartney stated to the Medical Section of the British Association, at their recent meeting, that he had been induced to try the effects of metallic ligatures, from observing that such substances frequently remained in the body without exciting any uneasiness, whilst the ordinary ligature fails from the injury and consequent inflammation excited by it. On applying ligatures of leaden wire to the arteries of dogs, he found, after death, that they remained, *in situ*, without surrounding inflammation, or were removed by intestinal absorption, the arteries being impervious. The same results were observed when the experiment was made on the jugular vein of rabbits. An improvement was made by Mr. Weiss on the leaden ligature, by substituting soft metal wire, capable of being knotted.

Long since the late Professor Physick suggested the use of leaden ligatures; and eleven years ago a number of experiments were tried with them by Dr. H. S. Levert, of Alabama, and an account of them was published in the No. of this Journal for May, 1829.—*Amer. Journ. of Med. Sciences*, for November.

